# Effect of Intermittent versus Continuous Light Exposure on Pupillary Light Response, As Evaluated by Pupillometry

**DOI:** 10.3389/fneur.2017.00746

**Published:** 2018-01-16

**Authors:** Shakoor Ba-Ali, Henrik Lund-Andersen, Hamid Ahmadi, Adam Elias Brøndsted

**Affiliations:** ^1^Department of Ophthalmology, Rigshospitalet, Glostrup, Denmark; ^2^Faculty of Health and Medical Sciences, University of Copenhagen, Copenhagen, Denmark

**Keywords:** pupillometry, post-illumination pupillary response, melanopsin, intrinsic photosensitive retinal ganglion cells, pupillary light response, rods and cones

## Abstract

**Objective:**

Continuous and intermittent stimuli with green light affect the pupillary light response (PLR) differently. Since the majority of pupillometric studies use blue and red lights, we investigated the effect of continuous and intermittent stimulations on the PLR using red and blue lights.

**Methods:**

Seventeen healthy subjects underwent continuous- and intermittent light stimuli, using red (643 nm) and blue light (463 nm). To avoid the influence of pupil size on the amount of light entering the eye, the procedures were repeated with the stimulus–eye in dilated condition. The maximal pupillary constriction and the early redilation phase of post-illumination pupillary response (PIPR_Early_) represented the mixed response of melanopsin and rod–cone photoreceptors. The late redilation phase of PIPR (PIPR_Late_) was the marker of melanopsin-containing retinal ganglion cells.

**Results:**

Intermittent stimuli with blue light elicited significantly larger maximal contraction during dilated condition (*P* = 0.001), and larger sustained pupillary contraction under dilated as well as undilated condition (*P* < 0.001) compared to continuous light exposure. Except the PIPR_Early_ during undilated condition, none of the PIPR metrics were significantly different between intermittent and continuous blue light stimuli. Intermittent red light stimuli elicited also a more sustained pupillary contraction regardless of mydriatic instillation (*P* ≤ 0.02). In addition, intermittent red light exposure resulted in a slightly larger PIPR_Early_ under undilated condition (*P* = 0.02) and a slightly larger PIPR_Late_ under dilated condition (*P* = 0.049). Except the PIPR_Late_ to continuous red light stimulus, all PIPR parameters were larger when the light was presented after induction of unilateral mydriasis.

**Conclusion:**

PLR parameters during and after light exposures depend on both the light stimulation mode and the entrance pupillary size.

## Introduction

Since the discovery of the melanopsin-containing intrinsic photosensitive retinal ganglion cells (ipRGCs) and their involvement in the pupillary light response (PLR), a common method for *in vivo* quantification of the ipRGCs response has been the measurement of PLR to different narrowband light stimuli. This method is popularly known as chromatic pupillometry. Based on the maximum spectral sensitivity and light intensity threshold of the different photoreceptors, various chromatic pupillometry protocols, aiming to differentiate the PLR driven by rods, cones, and melanopsin, have emerged (1–3). Chromatic pupillometry may potentially be used clinically as a quick and non-invasive method in the evaluation of retinal diseases. However, several challenges are related to the existing pupillometry protocols, including the lack of consensus on the stimulation mode.

The ipRGCs are a subset of retinal ganglion cells, which control the PLR (4). In fact, ipRGCs are the conduit for both the intrinsically melanopsin-mediated and the indirectly rod–cone-elicited PLR (4–6). Whereas the rod–cone activation produces a fast PLR, the melanopsin-induced electrophysiological response is much slower (7, 8). The sluggish intrinsic response of ipRGCs is observed as a sustained pupillary constriction long after stimulus offset, which is termed post-illumination pupillary response (PIPR) (7, 9). Recent studies have shown that PIPR has two phases: an early redilation phase, owing to mixed signals from ipRGCs and rod/cone, and a late redilation phase, which is solely maintained by the melanopsin contribution (10–12).

The light conditions differ between pupillometric studies, which lead to differences in both the PLR during light exposure and the PIPR. Previous work by Gooley et al. showed that stimulation with intermittent green light elicits greater pupillary contraction compared to continuous light exposure (9). By inserting short pulses of darkness during pupillary illumination (intermittent light stimuli), we expected to avoid the pupillary escape, i.e., pupillary dilation during continuous light stimulus (13). In the current study, we tested whether continuous and intermittent blue- and red light stimuli have different effects on PLR. In addition, we assessed the influence of unilateral mydriasis on the pupillary constriction and dilation.

## Materials and Methods

### Subjects

Seventeen healthy subjects, 12 females and 5 males with visual acuity ≥1.0 on the Snellen chart, were recruited from the Copenhagen area between April 2017 and September 2017 to the Department of Ophthalmology, Rigshospitalet, Denmark. The exclusion criteria were refractive error >6 diopters, color vision deficiency, use of any prescription medication, pregnancy, and present and past ocular or systemic diseases, which could potentially affect the PLR.

All participants underwent full ophthalmological examination including slit lamp biomicroscopy, indirect fundoscopy, intraocular pressure measurement (Goldmann tonometry), visual acuity test (Snellen chart), and color perception test (Ishihara 38 plates). Imaging procedures of retina included spectral-domain optical coherence tomography (Spectralis, Heidelberg Engineering GmbH, Heidelberg, Germany) and color fundus photography (Topcon Retinal Camera 50DX, Topcon Corporation, Tokyo, Japan).

Informed written consent was obtained from all subjects prior to the study attendance. The study was performed in accordance to the tenets of Helsinki declaration and was approved by the local committee on health research ethics in Denmark (protocol number: H-15013160).

### Pupillometry

We used a dual-channel chromatic pupillometer (DP-2000 Human Laboratory Pupillometer, NeurOptics, CA, USA), consisting of a compact stimulation and recording unit for each eye, thus permitting monocular or binocular dichoptic stimulation of the central field.

All subjects underwent intermittent and continuous light stimulation with the stimulated eye in non-mydriatic condition and after 30 min, with the stimulated eye in mydriatic condition, i.e., in total four measurements. The right eye was the dilated and stimulated, and the left eye was recorded for pupil-metrics analysis. However, in five patients, due to ocular abnormality confined to the right eye, the left eye was dilated. Unilateral mydriasis was induced by instillation of 10% phenylephrine hydrochloride and 1% tropicamide. The reason for pupil dilation was to ensure an equal of amount of light entering the eye through the pupil during intermittent and continuous light stimuli. Each protocol started with 5 min of dark adaptation, followed by the illumination of the right eye with red light (633 nm, 100 lx) and hereafter blue light (463 nm, 100 lx). The illuminance of red light corresponded to 300 CD/m^2^, whereas the blue light illuminance corresponded to 332 CD/m^2^. There was a 5 min pause in darkness between the red and blue light illumination. The resting time between continuous and intermittent light exposure protocols was minimum 30 min.

**Figure 1:**
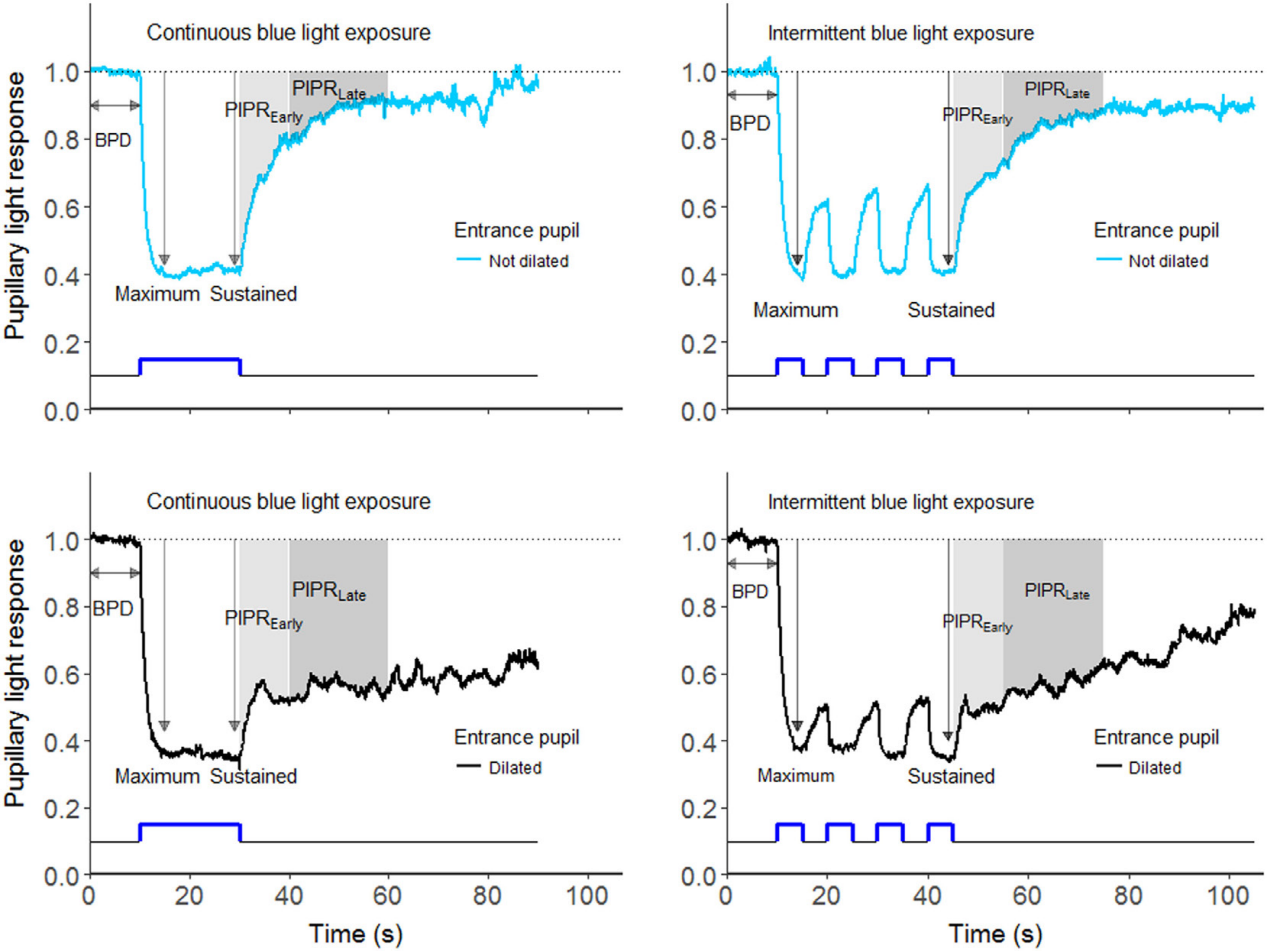
The pupillary light responses (PLR) elicited by continuous (left panel) and intermittent (right panel) blue light exposure (463 nm) in a representative healthy subject. In addition, the upper panel shows the PLR with the entrance pupil in undilated condition, whereas the lower panel shows the PLR with the entrance pupil in dilated condition. The outcomes of the study were baseline pupil diameter (BPD), maximum pupil contraction, early redilation phase of post-illumination pupillary light response (PIPR_Early_), and late redilation phase of PIPR (PIPR_Late_).

**Figure 2:**
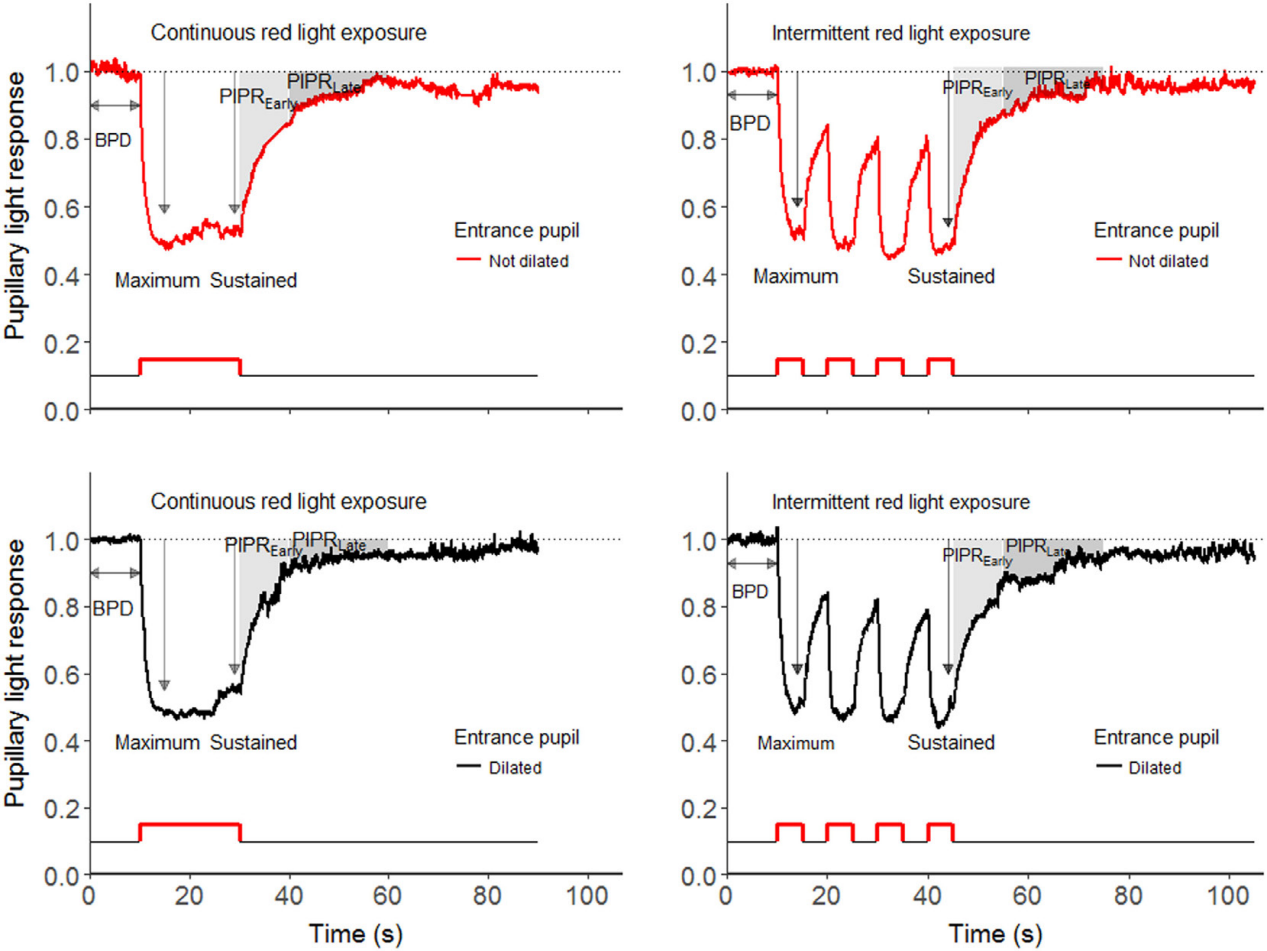
The pupillary light responses (PLR) elicited by continuous (left panel) and intermittent (right panel) red light exposure (633 nm) in a representative healthy subject. The pupil of the stimulus–eye is not dilated in the upper panel, whereas in the lower panel the pupil dilated. The outcomes of the study were baseline pupil diameter (BPD), maximum pupil contraction, early redilation phase of post-illumination pupillary light response (PIPR_Early_) and late phase of PIPR (PIPR_Late_).

The protocol with continuous stimulation, described in detail by Herbst et al. (14), consisted of an initial baseline pupil diameter (BPD) recording in 10 s of darkness, followed by 20 s continuous stimulus with blue or red light, and pupil diameter measurement in 60 s after the stimulus offset (Figures 1 and 2).

The protocol with intermittent light exposure started with BPD measurement in 10 s, followed by four sequences of 5 s light-on and 5 s light-off, and after the offset of the fourth stimulus the pupils were recorded in 60 s in the dark. This protocol was comparable to the protocol developed by Gooley et al., showing enhanced cone mediated PLR to intermittent green light exposure (9).

### Outcomes

The pupil diameter was normalized by dividing the actual measured pupil diameter in millimeter at any given time point by the mean BPD also in millimeter, yielding a relative pupil size. The pupillary metrics during light stimulation and the outcomes after light termination are described in Table 1.

**Table 1:** Definition of the pupillary metrics.

Pupil metrics	Definition
BPD (mm)	The average pupil diameter during the initial 10 s prior to light exposure

Maximal pupil contraction	The mean of maximum pupil contraction amplitude during 3–5 s of illumination time (15, 16)

Sustained pupil contraction	Pupil contraction amplitude during the last second of light exposure in order to assess pupillary escape

PIPR_Early_	The mean of pupil contraction amplitude from *t*_1_ = 0 s to *t*_2_ = 10 s after light offset with a pupillometry sampling rate at 30 Hz (15, 16): $\text{PIPR}=\frac{1-\sum_{{\text{t}}_{1}}^{{\text{t}}_{2}}\left(\text{normalised pupil diameter}\right)}{\left({\text{t}}_{2}-{\text{t}}_{1}\right)\times 30\text{Hz}}$

PIPR_Late_	The mean of pupil contraction amplitude from 10 to 30 s after the light exposure (15, 16)

*BPD, baseline pupil diameter; PIPR, post-illumination pupillary light response; PIPR_Early_, early phase redilation of post-illumination pupillary light response; PIPR_Late_: late redilation phase of post-illumination pupillary light response*.

The primary outcomes were the early and late redilation phases of PIPR, Figure 1. PIPR was calculated as the mean difference between normalized baseline pupil size (i.e., one) and the actual pupil size after light-offset (please see the shaded area in Figures 1 and 2).

### Statistics

Data analysis was performed using the statistical software package R, version 3.2.0. For all outcomes, mean ± SD values were reported. Due to repeated measured design, we used a linear mixed-effects model to compare the outcomes of continuous light stimuli to pupillometric outcomes attained with intermittent light exposures. To adjust for the pupillary dilation during light-off pulses of intermittent stimuli, we repeated pupillary measurements with the stimulated eye in dilated condition. So, in total we performed three comparisons: first, the pupillometry outcomes of intermittent light stimuli were compared with the outcomes attained by continuous light exposure. Next, the outcomes were compared with each other after that intermittent and continuous light were presented to dilated pupil. Third, the pupillometry outcomes attained in undilated condition was compared versus the outcomes measured after pupil dilation.

## Results

The mean age of our study population was 66 years (range, 57–78 years), the mean BMI was 25 ± 3 kg/m^2^, and the mean visual acuity was 90 ± 4 ERDRS letters, Table 2.

**Table 2:** The clinical profile of healthy controls.

Demographic parameters	Mean ± SD
Sex (female:male)	12:5
Age (years)	65.9 ± 6.3
BMI (kg/m^2^)	25.1 ± 3.5
Visual acuity (ETDRS)	90.4 ± 4.4
IOP (mmHg)	15.6 ± 2.8

The BPD prior to both continuous- and intermittent blue light exposures was not significantly different from each other, neither in undilated condition nor when the consensual pupil was dilated, Table 3. Similarly, there was no significant difference between BPD prior to the two stimulation types, when we used red light. The maximal pupil contraction was significantly larger during intermittent blue light stimulation in dilated condition (*P* = 0.001), but not in undilated condition (*P* = 0.13). However, maximal pupil contraction to intermittent red light exposure was not significantly different from maximal pupil contraction to continuous light stimuli, regardless of baseline pupil size. In undilated condition, the intermittent blue light stimuli produced both larger sustained pupil contraction (*P* = 0.0005) and larger PIPR_Early_ (*P* = 0.02). Similarly, red light exposure in intermittent form elicited also larger sustained pupil contraction and PIPR_Early_ (*P* = 0.02). The PIPR_Late_ of the consensual eye was not significantly different following intermittent and continuous blue light exposures when the stimulated eye was dilated (*P* = 0.77). However, intermittent red light stimuli produced significantly larger PIPR_Late_ in dilated condition (*P* = 0.049).

**Table 3:** Effect of continuous versus intermittent light stimuli on the pupillary light response (PLR).

	Undilated pupil			Dilated pupil		
	Continuous	Intermittent	*P*	Continuous	Intermittent	*P*
**Blue light**						
BPD (mm)	6.2 ± 0.5	6.3 ± 0.6	*0.27*	6.2 ± 0.5	6.2 ± 0.5	*0.83*
Maximal pupil contraction	0.59 ± 0.0	0.58 ± 0.0	*0.13*	0.61 ± 0.0	0.65 ± 0.0	*0.001*
Sustained pupil contraction	0.58 ± 0.0	0.64 ± 0.0	*0.0005*	0.60 ± 0.0	0.66 ± 0.0	<*0.0001*
PIPR_Early_	0.33 ± 0.1	0.37 ± 0.1	*0.02*	0.51 ± 0.0	0.53 ± 0.0	*0.11*
PIPR_Late_	0.15 ± 0.1	0.19 ± 0.1	*0.07*	0.48 ± 0.1	0.48 ± 0.1	*0.77*

**Red light**						
BPD (mm)	6.3 ± 0.6	6.3 ± 0.6	*0.52*	6.2 ± 0.6	6.3 ± 0.5	*0.18*
Maximal contraction	0.52 ± 0.1	0.50 ± 0.0	*0.19*	0.51 ± 0.0	0.51 ± 0.1	*0.79*
Sustained contraction	0.44 ± 0.1	0.47 ± 0.1	*0.02*	0.51 ± 0.0	0.51 ± 0.1	*0.002*
PIPR_Early_	0.19 ± 0.0	0.21 ± 0.0	*0.02*	0.22 ± 0.0	0.23 ± 0.0	*0.23*
PIPR_Late_	0.05 ± 0.0	0.05 ± 0.0	*0.92*	0.05 ± 0.0	0.07 ± 0.0	*0.049*

*Comparison of the consensual PLR parameters between continuous- and intermittent light stimuli. Pupillometry was performed both in undilated condition (left) and in dilated condition (right)*.

*BPD, baseline pupil diameter; PIPR_Early_, early redilation phase of post-illumination pupil response; PIPR_Late_, late redilation phase of post-illumination pupil response*.

**Figure 3:**
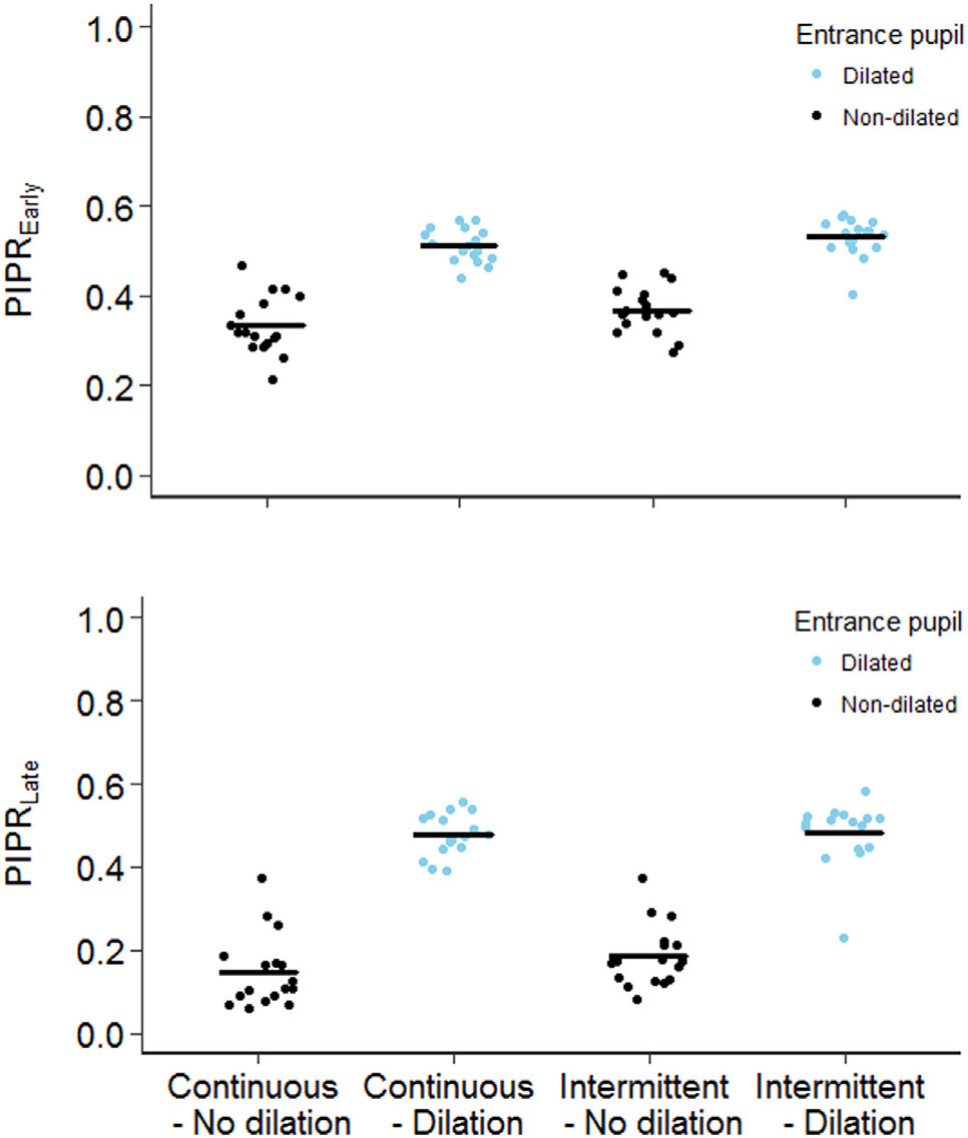
The early and the late redilation phases of post-illumination pupillary light response (PIPR_Early_ and PIPR_Late_) to continuous (left panel) and intermittent blue light (right panel), in dilated and undilated condition.

When we compared the pupil metrics in undilated condition versus outcomes after mydriatic instillation in the stimulated eye, all pupil metrics to blue light stimuli, except the BPD, were significantly larger after pupil dilation, Table 4. For example, our primary outcome, i.e., PIPR_Late_ following continuous blue light stimuli, increased by 225% when the pupillometry was performed after pupil dilation (*P* < 0.001), Figure 3. However, for the red light stimuli, only sustained pupillary contraction and PIPR_Early_ were significantly larger following dilation of the pupil exposed to light, Table 4.

**Table 4:** Effect of baseline pupillary size on pupillary light response (PLR).

	Continuous light stimuli (mean ± SD)			Intermittent light stimuli (mean ± SD)			
	Undilated pupil	Dilated pupil	*P*	Undilated pupil	*P*	Dilated pupil	*P*
**Blue light**							
BPD (mm)	6.2 ± 0.5	6.2 ± 0.5	*0.69*	6.3 ± 0.6	*0.27*	6.2 ± 0.5	*0.81*
Maximal pupil contraction	0.59 ± 0.0	0.61 ± 0.0	*0.03*	0.58 ± 0.0	*0.13*	0.67 ± 0.0	<*0.01*
Sustained pupil contraction	0.58 ± 0.0	0.60 ± 0.0	<*0.01*	0.64 ± 0.0	<*0.01*	0.66 ± 0.0	<*0.01*
PIPR_Early_	0.33 ± 0.1	0.51 ± 0.0	<*0.01*	0.37 ± 0.1	*0.02*	0.53 ± 0.0	<*0.01*
PIPR_Late_	0.15 ± 0.1	0.48 ± 0.1	<0.01	0.19 ± 0.1	*0.07*	0.48 ± 0.1	<0.01

**Red light**							
BPD (mm)	6.3 ± 0.6	6.2 ± 0.6	*0.09*	6.3 ± 0.6	*0.52*	6.3 ± 0.5	*0.36*
Maximal contraction	0.52 ± 0.1	0.50 ± 0.0	*0.16*	0.51 ± 0.0	*0.19*	0.51 ± 0.1	*0.20*
Sustained contraction	0.44 ± 0.1	0.51 ± 0.0	*0.02*	0.47 ± 0.1	<*0.01*	0.51 ± 0.1	<*0.01*
PIPR_Early_	0.19 ± 0.0	0.22 ± 0.0	*0.004*	0.21 ± 0.0	*0.02*	0.23 ± 0.0	*0.0001*
PIPR_Late_	0.05 ± 0.0	0.05 ± 0.0	*0.67*	0.05 ± 0.0	*0.92*	0.07 ± 0.0	*0.01*

*This table shows a comparison of the consensual PLR parameters before and after dilation of the pupil, stimulated with continuous and intermittent light stimuli*.

*BPD, baseline pupil diameter; PIPR_Early_, early redilation phase of post-illumination pupil response; PIPR_Late_, late redilation phase of post-illumination pupil response*.

## Discussion

The purpose of the current study was to compare the effects of intermittent and continuous light stimuli on the PLR metrics including the early and the late phases of PIPR. Moreover, we aimed to investigate the effect of entrance pupil size on pupillary constriction- and dilation parameters by inducing mydriasis in the stimulated eye and recording the consensual PLR. We found that sustained pupil contraction during both blue and red light stimuli was significantly larger when the lights were presented in intermittent form compared to continuous light stimuli, and this effect was present regardless of dilation of the entrance pupil size. Conversely, maximal pupil contraction to intermittent blue light stimuli was only larger when the light was presented to the dilated pupil, indicating an effect of the entrance pupil size. The early redilation phase of PIPR was also larger after intermittent blue light stimuli, but the effect diminished after induction of unilateral mydriasis. The melanopsin-mediated late redilation phase of PIPR to blue light was not changed with intermittent stimuli compared to continuous stimulus—regardless of entrance pupil size. However, intermittent red light stimuli after induction of unilateral mydriasis elicited a larger PIPR during the late redilation phase. Both pupillary constrictions during light exposure and PIPR parameters after light-offset were significantly larger with both continuous and intermittent light stimuli after that the entrance pupil was dilated.

Previous pupillometric studies have reported that after few seconds of light stimulation, the pupil begins to dilate despite continuous illumination and this relative dilation of the pupil under continuous illumination is termed “pupillary escape” (13). Pupillary escape reflects the kinetics of cones, which saturate after few seconds of constant illumination (9, 11). In our previous work, we showed a prolonged pupillary constriction during continuous blue light stimulus in choroideremia patients with severely rod–cone degeneration, indicating the lack of cone-mediated pupillary escape (11). Gooley et al. proposed intermittent light stimuli to allow cone desaturation during the short pulses of dark and consequently eliciting a larger pupillary constriction under illumination and a more sustained pupillary constriction after light-offset (9). The intermittent light stimuli stimulate mainly the cones, because cone photoreceptors adapt briskly to dark/light stimuli and desaturate during the short pulses of darkness between light exposures (7, 8). Accordingly, our findings in terms of larger sustained pupillary constriction and PIPR_Early_ to intermittent red light exposure may be caused by the cones. However, the larger pupillary constriction during the late phase of PIPR to intermittent *red light* exposure is surprising and in contrary to a study by Adhikari et al. reporting that PIPR ≥1.3 s after light cessation is entirely controlled by ipRGCs (10). Nevertheless, this finding is in agreement with the report by Kostic et al., showing that cones contribute to prolonged pupil constriction at 9.5 s after bright blue light stimulus (12). However, there are some discrepancies between our study and the two studies we just referred to. Both Adhikari and Kostic used single pulses of 1 and 0.5 s, respectively, which in comparison to the stimuli used in our study were much shorter. The reason for the longer stimulus duration in the current study was a previous work by Gamlin et al. reporting that the PLR, under pharmacological blockade of rod- and cone-input, is delayed by approximately 1 s, suggesting that 1 s may be insufficient to evoke the direct ipRGCs response (7). Moreover, Lall et al. showed that melanopsin-mediated PLR becomes clearly dominant after 0.8 s of light stimulation (17). Another difference was the stimulation type: both groups used single light stimuli, whereas in our study we used continuous and intermittent light stimuli. The results of Gooley et al. using intermittent stimuli were similar to our results (9). Hansen et al. showed also increased pupillary constriction with intermittent light stimuli (18).

Since both the intermittent green and the intermittent red light exposures stimulate cone photoreceptors, the current study provides additional evidence on the fact that cone’s contribution to the early and late phases of PIPR is dependent on the mode of light stimuli. Hence, intermittent light stimuli allow cones to dark adapt prior to the next light pulse and provide sequential synaptic input to ipRGCs for non-imaging purposes (8). The ipRGCs are the conduit for both the direct melanopsin-mediated light signals and the indirect rod/cone photic inputs. Whereas the indirect rod inputs and the direct melanopsin signal encode the average amount of light variations during low and higher irradiances, respectively, the ipRGCs uses the indirect synaptic input from cones to detect the short oscillation of ambient light, signaling small changes in light intensity to the brain for non-image forming processes (17, 19–21). Thus, we are hypothesizing that cone photoreceptors contribute to the late phase of PIPR; however, this contribution is happening mainly when the red light is presented as intermittent form. The latter is corresponding well to the fast kinetic of the cones and the peak spectral sensitivity of L-cones (22). Hence, the contribution of red light stimuli to the late phase of PIPR is much smaller compared to blue light exposure.

Another part of our study was to evaluate the effect of entrance pupil size on pupillometric parameters and we found that pupillary constriction both during illumination and after light offset was significantly larger after unilateral mydriasis of the entrance pupil. This effect was present for both continuous and intermittent light stimuli. A larger pupillary size allows a higher amount of light entering the eye, which activates a larger retinal area. Previous study (*n* = 10) has reported that entrance pupil size affects the magnitude of PIPR to blue light stimulus, but not to red light exposure (23). Other studies have reported that iris color influences pupillary constriction and redilation (24, 25). However, in our study we did not find any effect of iris color on the pupillary constriction, neither during illumination nor after light offset. Whether there is an effect of iris color or not, we recommend inducing unilateral mydriasis prior to retinal illumination in future pupillometric studies.

### Limitations

Pupil size can be affected by a range of factors, most of which in this study could be avoided by applying the two protocols on the same subjects. Although we investigated the subjects during the same time interval of the day, one cannot completely exclude the effect of arousal level on the pupil size and the pupil response (26, 27).

## Conclusion

Intermittent stimulations with both blue and red light evoked a larger sustained pupillary constriction compared to continuous illumination. Entrance pupil size affects both pupillary constriction during light exposure and the PIPR parameters after light-offset. Hence, we recommend unilateral mydriasis in future pupillometric studies.

## Ethics Statement

Informed written consent was obtained from all subjects prior to the study attendance. The study was performed in accordance to the tenets of Helsinki declaration and was approved by the local committee on health research ethics in Denmark (protocol number: H-15013160).

## Author Contributions

SB and HA contributed to data collection. SB and AEB performed the statistical analysis. SB, H. LA, and AEB contributed to the goal, design, and the whole concept of the project. All the authors read and edited the manuscript.

## Conflict of Interest Statement

The authors declare that the research was conducted in the absence of any commercial or financial relationships that could be construed as a potential conflict of interest.
